# Digital Health Strategies to Fight COVID-19 Worldwide: Challenges, Recommendations, and a Call for Papers

**DOI:** 10.2196/19284

**Published:** 2020-06-16

**Authors:** Guy Fagherazzi, Catherine Goetzinger, Mohammed Ally Rashid, Gloria A Aguayo, Laetitia Huiart

**Affiliations:** 1 Luxembourg Institute of Health Strassen Luxembourg

**Keywords:** coronavirus, COVID-19, digital health, eHealth, digital technology, health care, surveillance, communication, review, epidemiology, infodemiology, public health

## Abstract

The coronavirus disease (COVID-19) pandemic has created an urgent need for coordinated mechanisms to respond to the outbreak across health sectors, and digital health solutions have been identified as promising approaches to address this challenge. This editorial discusses the current situation regarding digital health solutions to fight COVID-19 as well as the challenges and ethical hurdles to broad and long-term implementation of these solutions. To decrease the risk of infection, telemedicine has been used as a successful health care model in both emergency and primary care. Official communication plans should promote facile and diverse channels to inform people about the pandemic and to avoid rumors and reduce threats to public health. Social media platforms such as Twitter and Google Trends analyses are highly beneficial to model pandemic trends as well as to monitor the evolution of patients’ symptoms or public reaction to the pandemic over time. However, acceptability of digital solutions may face challenges due to potential conflicts with users’ cultural, moral, and religious backgrounds. Digital tools can provide collective public health benefits; however, they may be intrusive and can erode individual freedoms or leave vulnerable populations behind. The COVID-19 pandemic has demonstrated the strong potential of various digital health solutions that have been tested during the crisis. More concerted measures should be implemented to ensure that future digital health initiatives will have a greater impact on the epidemic and meet the most strategic needs to ease the life of people who are at the forefront of the crisis.

## Background

Countries around the world have been affected by the COVID-19 pandemic since December 2019 [1], and the health care systems in these countries are rapidly adapting to the increasing demand. The World Health Organization (WHO) has called for coordinated mechanisms to support the response to the outbreak across health sectors, and digital health solutions have been identified as one of the most promising approaches to address this challenge in modern societies [2].

The COVID-19 pandemic is singular in many ways. First, in terms of number of people infected, transmissibility, and spectrum of clinical severity, it has had a greater impact to date than previous epidemics such as pandemic influenza, Middle East respiratory syndrome (MERS), severe acute respiratory syndrome (SARS), or Ebola virus [3]. Second, COVID-19 can be considered as the first true global epidemic of this magnitude in the digital era; digital health solutions, which have reached a certain level of maturity but are not widely deployed and accepted yet, can play a major part in our response to the crisis [4]. Indeed, the COVID-19 pandemic is occurring in an era of massive technological advancement. Digital tools can effectively support institutions during a pandemic by facilitating the immediate widespread distribution of information [5], tracking transmission in real time, creating virtual venues for meetings or day-to-day operations, and providing telemedicine visits for patients [6]. However, some digital health strategies and tools may face challenges associated with barriers to access, acceptability, and ethical issues. For instance, some governments worldwide are responding to this public health emergency with an unprecedented array of surveillance tools designed to identify and track people who may be infectious [7]. These measures have escalated existing debates regarding individual privacy and government oversight of private citizens.

The COVID-19 pandemic is providing a starting point to discuss how digital health solutions can and should be leveraged to address this unprecedented crisis [8]. This editorial offers an overview of the digital health strategies that have been employed worldwide to fight COVID-19 to date as well as a discussion of the challenges to developing more meaningful [9] and ethical solutions in the near future.

## Digital Health Care Models

**Figure 1:**
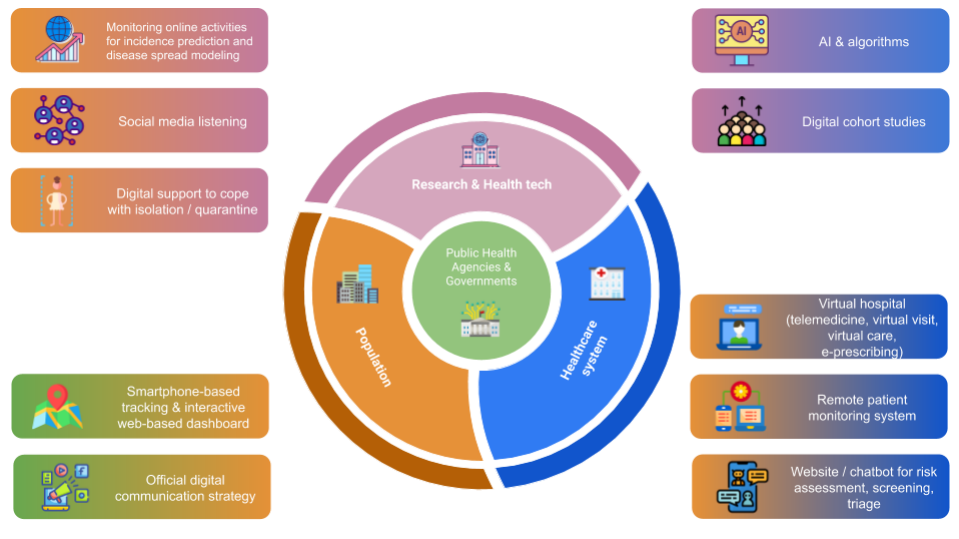
The COVID-19 digital health ecosystem. AI: artifical intelligence; e-prescribing: electronic prescribing; tech: technology.

New health care models are needed during the COVID-19 pandemic. Due to the high transmission rate of COVID-19, most countries have ordered strict lockdowns; hence, patient-physician communication and visits are challenging. In the era of digital health technologies, the focus on new models has shifted to telehealth (virtual visits, virtual care), mobile apps (remote patient monitoring), and websites and chatbots (risk assessment, screening, triage). Figure 1 describes the main stakeholders at the forefront of the COVID_19 crisis (the population, the health care systems, and the research and health technology environment in coordination with public health agencies or governments) with significant illustrations of efficient digital health strategies that have been implemented to date at various levels.

## Telemedicine and Remote Patient Monitoring Systems

Telemedicine allows patients to receive care at home [10], thus avoiding the spread of COVID-19 in overcrowded emergency or waiting rooms [11]. Telemedicine not only supports secured care for COVID-19 patients but also allows routine primary care and electronic prescriptions (e-prescriptions). In addition, health care professionals can screen and monitor symptoms in real time, provide useful medical advice when needed, and keep stable patients at home away from overloaded hospitals [12].

Additionally, these tools can serve as useful resources to rapidly collect meaningful information on large cohorts of patients to study the evolution of their symptoms in real time; this increases understanding of the different clinical phenotypes of people infected with COVID-19 and enables the study of its long-term health consequences. Such a prospective cohort was recently established in Luxembourg (Predi-COVID) to study factors associated with COVID-19 disease severity; this study uses data from the national telesurveillance system that is used for virtually all patients who tested positive for COVID-19 and combines the data with biological sampling, electronic patient-reported outcomes, and innovative digital data collections such as smartphone-based voice recording to identify vocal biomarkers of respiratory syndromes. In another study, Wang et al [13] described armband sensors that can remotely measure the accuracy of hand hygiene based on WHO procedures.

## Triage and Risk Assessment

**Figure 2:**
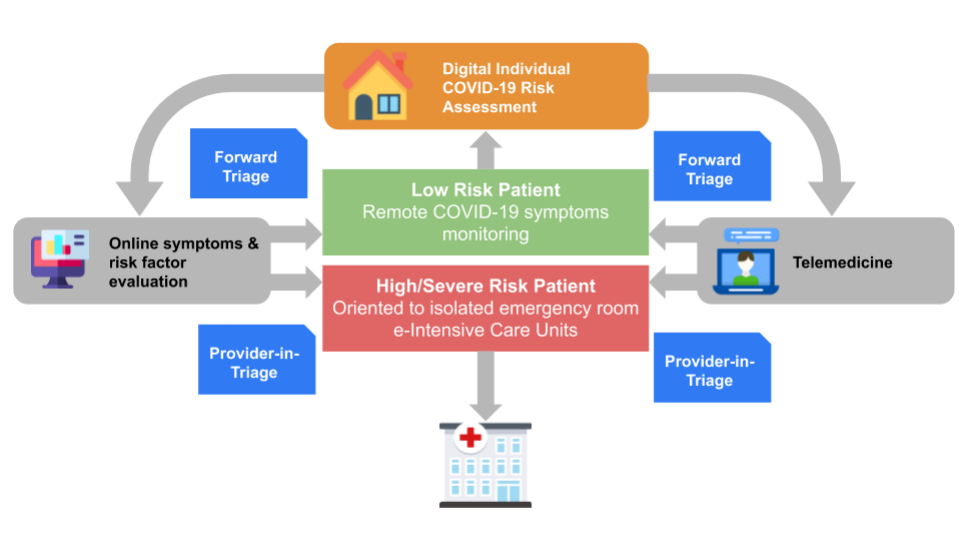
Risk assessment and triage of COVID-19 in a digital health care model. COVID-19: coronavirus disease.

The COVID-19 virus has caused panic and uncertainty within populations; to ensure better allocation of resources, telemedicine based on videoconferences can be used not only to comfort patients but also for triage [14]. Hollander et al [15] highlighted that telemedicine can serve as a triage system in different phases. The first stage is “forward triage;” here, patients are categorized as possibly COVID-19 infected or uninfected before they arrive at the hospital. In addition, telemedicine ensures close monitoring of less severely infected patients at home by allowing communication around the clock. The second phase of triage occurs in the hospital and is referred to as the “provider-in-triage model.” In this phase, patients at high risk for COVID-19 are screened by rapid evaluation of clinical characteristics and testing. Patients are kept in isolated emergency rooms, where they are given tablet devices to communicate with professionals [15]; the tablets are cleaned between patients. To reduce risk of infection among health care professionals, hospitals have also implemented electronic intensive care unit monitoring. For example, a few countries have launched within-hospital telemedicine programs. A robot enters the room of a patient infected with COVID-19 to monitor both vital and cardiac signs and enable communication between the patient and health care professionals where applicable. All the robot’s actions are controlled by nurses or physicians outside the room [16]. Figure 2 shows the typical path of an individual from the time they have questions about their health or symptoms potentially related to COVID-19. Digital technologies now enable online symptom checks, video consultations, and e-prescriptions and allow forward triage instead of overloading hospitals and increasing the spread of the virus. Low-risk patients can be regularly monitored from home, whereas patients with more severe symptoms can be monitored at hospitals with a provider-in-triage using digital tools.

Web-based symptom trackers such as maladiecoronavirus.fr [17] in France and beatcovid19now.org [18] in Australia were rapidly established to help individuals assess their risk for COVID-19; these trackers are being updated as knowledge of COVID-19 evolves. Site users must answer several questions to feed the risk prediction algorithms; then, based on risk stratification, the website advises the user to call a hotline to receive further instructions or to stay at home and follow international guidelines. Similarly, chatbots such as Symptoma are systems that digitally assist individuals in performing risk assessment of COVID-19 [19].

## Digital Communication During the Pandemic

**Figure 3:**
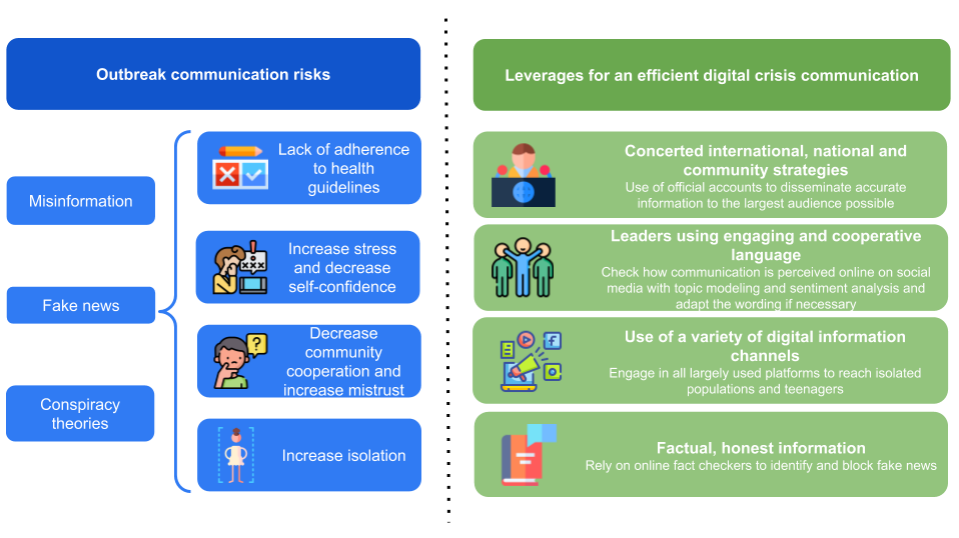
Communication risks and strategies during disease outbreaks.

Communication between political leaders and scientific authorities is crucial during a pandemic crisis [20] (Figure 3). Since the start of the COVID-19 pandemic, disinformation, “fake news,” and conspiracy theories about the origin, spread, and treatment of the disease have been present on the internet and in government communications, social media, and SMS text messages. These communication problems also cause confusion and mistrust; the consequences are negative at both the individual and community levels. To provide the most useful information in a timely fashion, effective digital risk communication has been established internationally by WHO as well as nationally and regionally through an active factual information campaign using several digital channels. Good communication strategies help manage people's fear and increase the likelihood that they will adhere to the measures imposed on them during the crisis. Risk communication promotes community engagement, avoids or decreases the profusion of rumors, and reduces threats to public health. However, poor risk communication can create a great deal of confusion, frustration, and future mistrust of leaders. Communication in epidemics must not only be clear and unambiguous but must also reach the target population quickly (health workers, local authorities, and the public). For instance, guidelines around practicing social distancing have been judged to be unclear and prone to misunderstanding. These guidelines should be followed with more direct messages, such as “stay at home,” especially to clarify the concept of social distancing for vulnerable populations or younger people. The WHO webpage uses the phrase “physical distance” in addition to “social distance” to emphasize this necessity. However, social contact must be stimulated, especially for older persons [21]. Digital methods appear to be promising solutions, as they enable people to maintain physical distance while keeping informed or maintaining contact with others; for example, the WhatsApp messaging platform provides health alerts in different languages [22].

In addition, digital health can play a major role at a governmental level. Government initiatives have been taken to provide guidelines and information on government websites to inform the population about appropriate behavior during the lockdown period, hand hygiene, etc. Most of these initiatives were supported by international guidelines from WHO or the US Centers for Disease Control and Prevention (CDC).

The use of apps or instant messaging services to enable ministries or health agencies to contact health workers remotely and to inform government officials about the number of cases, symptoms, and prevention measures are just a few examples of measures that have been efficiently used in previous pandemics [23] or early in the COVID-19 crisis [22]. A crucial consequence of a bad risk communication strategy is the likelihood of increased levels of anxiety within the population. Contradictory information and overexposure to sensationalist headlines from mass media and social media can affect people’s mental health and increase the general level of fear. Lockdown can amplify the problem due to increased exposure to social media and rumors as well as lack of confrontation of ideas and debate in the real world [24]. Consequently, lockdown reinforces the importance of institutional development of digital strategies. Therefore, during the outbreak, official communication plans should promote easily accessible and diverse channels of digital communication at all stages and for different purposes (global, regional, and community-based communication). These channels should be integrated as an information system, as a communication tool, and for detecting and supporting people at risk [25]. An example of a communication strategy that was developed very quickly and effectively to fight fear and rumors among a population is the sending of messages by the government of Singapore through WhatsApp [26]. Finally, a strategy to include marginalized and vulnerable populations should be planned. Such a strategy should involve establishing multiple means of communication, including digital platforms; using adequate, simple, and nondiscriminatory language; and taking differences in health literacy into account [27].

## Digital Data to Model COVID-19 Spread, Evolution, and Perception

The monitoring of online or social media activities for public health purposes has been investigated since the first days of the digital epidemiology field in the early 2010s, with the objective of capturing health-related trends and modeling disease outbreaks. The most famous examples of internet health surveillance were developed to predict the incidence of influenza, such as Google Flu Trends, or to obtain insights from social media platforms such as Twitter about influenza A/H1N1 [28], measles [29], the Zika virus [30], and the Ebola virus [31,32]. Despite their high potential in public health, these studies have been criticized for their lack of theorization and appropriate standard methodology [33], which can prevent comparison of their results and raises questions regarding the safety of relying on their findings to design targeted public health measures in the real world. To move beyond the simplistic thought that Twitter is a “giant playground” for data scientists, ongoing initiatives to use Twitter data to study the COVID-19 outbreak should build upon previous mistakes, with a more rigorous approach as well as an open source/open data mentality.

Online activities are very good indicators of trends in society. Exploiting data such as Google Trends [34], Wikipedia searches, or the Baidu Index and Weibo Index in China has already proved useful. In China, internet searches and social media data related to COVID-19 have been shown to be highly correlated with daily disease incidence and exhibit an online peak 10 to 14 days before the peak of daily incidences in the real world [35]. Although there are few reports to date, such data sources could also prove useful for early identification of less common symptoms related to COVID-19 using a similar approach to that used to detect weak pharmacovigilance signals from social media and identify side effects of drugs [36]. The main advantage of these data sources is that they can be accessed early in the epidemic and at low cost.

However, predicting disease incidence from online activities or social media data has never been used in a real time prospective fashion for public health guidance on a large scale; rather, such predictions are performed retrospectively by training algorithms to replicate gold-standard data, such as those obtained from the CDC [37], as closely as possible. COVID-19 may be the first outbreak where such a prospective analysis occurs, combining data from standard sources of disease surveillance with social media data or activity trackers [38] to improve overall accuracy.

Social media data analysis is also relevant during the COVID-19 outbreak to monitor how people react to the pandemic evolution over time (sentiment, anxiety, level of stress) as well as common beliefs, opinions [39,40], fears, or hopes regarding treatment or vaccines. Perhaps most importantly, identifying and combating the spread of “fake news” about COVID-19 is highly necessary in our modern digital society [41,42]. This could help to rationalize the debates and actions around COVID-19 and should be further extended [43].

## Discussion

### Challenges Related to Digital Health Implementation

The COVID-19 crisis is a typical example of the impossibility of establishing a single global technological solution to a given problem. To increase acceptability of digital technologies, the different cultural, moral, and religious backgrounds of users should be considered. With priority given to collective public health benefit and maintaining local social order during the current emergency [44], digital measures can be intrusive and can erode individual freedoms. In some countries, a strong digital divide persists today, and vulnerable populations may be overlooked during the implementation of digital approaches [45-47]. Digital solutions may be less frequently understood and used by people with low health literacy levels or by specific subgroups such as minorities, older individuals, or people who live in rural or low-income areas [48]. For all these reasons, digital approaches can be received in very different ways when they are applied in high-income, medium-income, or low-income areas or when they are deployed in individualistic versus collectivistic countries [49].

In Singapore, an app called TraceTogether has been used to track patients infected with COVID-19. Based on Bluetooth signaling, Singaporean health authorities can track individuals and inform them if they have been in contact with a patient infected with COVID-19 [50]. This patient monitoring system may be beneficial in disease surveillance and outbreak management; however, in European countries, it would not be possible to use it due to individual data protection laws. France conceived the CoronApp, which is based on similar standards [51]. Individuals need to register on the app and provide information on their health status and whether they have symptoms. A geolocation system is used to trace this information and is updated every hour. King's College London conceived the COVID Symptom Tracker app, which aims to help patients monitor their own symptoms [52]. The app gained such popularity that it is now also used in the United States. In addition to its individual benefits, the app can be used for research purposes to study the epidemiology of COVID-19.

Urged by the European Commission, an unprecedented consortium of eight giant telecommunications companies recently agreed to share aggregated and anonymized geolocation data from their clients to track their movements and activities. This raises numerous questions around data privacy [53] and ethics in Western and African countries [54], despite the availability of privacy-preserving proximity tracing technology such as the Decentralized Privacy-Preserving Proximity Tracing (DP-3T) protocol [55]. In Asia, the response to the COVID-19 crisis in South Korea has received much attention and has been widely cited as an exemplary model. With the increased flexibility in South Korea’s digital privacy laws since the MERS crisis in 2015, numerous aggressive digital solutions have been used, such as a national smartphone app, GPS tracking of infected people, and detailed emergency alerts when a positive case has been identified in a person’s geographical area. However, it was proved that this approach was only efficient to reduce the number of new daily cases due to a vast parallel testing campaign to identify COVID-19 hotspots combined with strong isolation and quarantine measures. In Europe, where testing is not as widespread as in South Asia, the use of such monitoring using GPS coordinates is therefore ethically questionable.

Regarding telehealth, the challenge of implementing feasible systems lies with governments in most cases [56], as billing systems must be adapted [15]. Telemedicine consultations must also ensure patient security in terms of data protection. Hence, close collaborations between different actors, such as health care professionals, health technology companies, and health politicians, must be ensured [15]. Once such a system is running, both patients and health care professionals can benefit from better allocation of resources and adequate care, all with reduced risk of exposure. Telemedicine and digital technologies such as apps can also play larger roles in the present crisis not only to fight COVID-19 but also to address the frequent health issues associated with isolation or quarantine, such as psychological needs [57], mental health conditions [58], and physical inactivity [59]. Lastly, some developing countries face major obstacles to the effective delivery of digital health solutions in rural and remote locations, such as incomplete or insufficient basic digital infrastructures (eg, computers, internet networks, and electricity), lack of sustainable funding to develop, operate, and maintain digital platforms, and high telecommunication costs [4,60,61].

### Recommendations to Improve Future Digital Health Systems

In France, where the COVID-19 epidemic is growing at a rapid pace, the health technology ecosystem has rapidly and generously contributed to management of the crisis. Numerous telemedicine and telemonitoring platforms have been locally deployed in hospitals or general practices. However, a lack of coordination between operators has led to issues such as an absence of consensus about the symptoms to monitor and the alert systems to establish. We could argue that some telemonitoring is better than no monitoring; however, given the rapidly evolving body of knowledge around COVID-19, more transparent communication and greater comparability should be pursued to rapidly and homogeneously update the different solutions. Otherwise, some populations will lose the opportunity to gather this data and later use it for research or evaluation.

In previous emergency situations such as the Ebola virus outbreak, Hurricane Harvey, and Hurricane Irma, as well as currently during the COVID-19 pandemic, telehealth has been explored to support patient-physician communication and, hence, improved health outcomes. It must be ensured that outside of emergency situations, telehealth will be appropriately implemented into national health care systems in the long term. This would facilitate use of telehealth systems during outbreaks [62]. Germany is an example of a country where early implementation of telemedicine and mobile apps into the health care sector since 2018 have shown short-term benefits during the COVID-19 pandemic [63]. The German health ministry even claims that they now aim to accelerate the digitization of health care in Germany and help to translate new laws into practical solutions.

Digital initiatives to fight COVID-19 should be relevant to both hospital and public health systems, adapted to the population, rapidly deployable, and capable of evolving with the growth of the body of knowledge related to COVID-19. Regarding common standards for data collection in digital COVID-19 solutions, future initiatives should consider the example of ISARIC (the International Severe Acute Respiratory and emerging Infection Consortium [64]), who successfully organized clinical research on COVID-19 by providing standardized but customizable clinical research forms in several languages, ensuring a harmonized core set of international and comparable data, or the DP-3T initiative, an open implementation of a decentralized privacy-preserving proximity tracing solution [65].

### Conclusion

Time is key to fight COVID-19, and digital health solutions provide the opportunity to buy time and human resources. As the COVID-19 pandemic is the first true global health crisis in the digital era, we have observed and will observe a plethora of digital solutions. This pandemic has at least demonstrated the usefulness and reactivity of digital health solutions and constitutes an opportunity to insert these solutions into our health care systems in the long term. This creates an urgent need for policy makers, researchers, and health professionals to collectively and efficiently implement digital solutions into practice without further fragmenting the existing landscapes of care. We now call for more concerted measures to have an optimal impact on the epidemic and to address the most strategic needs to ease the life of people who are at the forefront of the COVID-19 crisis [66].

## Abbreviations

CDC: US Centers for Disease Control and Prevention
COVID-19: coronavirus disease
DP-3T: Decentralized Privacy-Preserving Proximity Tracing
e-prescription: electronic prescription
ISARIC: International Severe Acute Respiratory and emerging Infection Consortium
MERS: Middle East respiratory syndrome
SARS: severe acute respiratory syndrome
WHO: World Health Organization
